# Street Pavements

**Published:** 1901-09-14

**Authors:** 


### EDITOR'S LETTER-BOX.

[Our Correspondents are reminded that prolixity is a great bar to publication, and that brevity of style and conciseness of statement greatly facilitate early insertion.]

### STREET PAVEMENTS.

REFERRING to certain strictures on various forms of wood paving which appeared in a recent article in THE HOSPITAL, "Audi alteram partem" writes:—

"Although the great disadvantages pointed out do exist certainly in the cases of deal and other what are known as soft woods, these disadvantages do not obtain where the West Australian hard woods—notably jarrah (eucalyptus marginata) and karri (eucalyptus diversicolor)—are employed for paving thoroughfares, and they are employed for that purpose both in London and the provinces on a very extensive scale indeed, with the most successful results. Moreover, it is most important to note from a sanitary point of view that one of the principal advantages of the eucalyptus woods of Australia for street-paving purposes lies in their anti-septic and non-absorbent character. Objections from a sanitary point of view, which have been urged against wood paving do not apply to these woods, if they are kept fairly well cleansed, and if, particularly in very dry weather, they are properly watered."

